# Nanoparticles Based on Chitosan as Carriers for the Combined Herbicides Imazapic and Imazapyr

**DOI:** 10.1038/srep19768

**Published:** 2016-01-27

**Authors:** Cintia Rodrigues Maruyama, Mariana Guilger, Mônica Pascoli, Natalia Bileshy-José, P.C. Abhilash, Leonardo Fernandes Fraceto, Renata de Lima

**Affiliations:** 1 Laboratory of Biotechnology, University of Sorocaba, Rodovia Raposo Tavares, km 92, 18023-000, Sorocaba, Brazil; 2 Department of Environmental Engineering, São Paulo State University (UNESP), Sorocaba, Brazil; 3 Federal University of São Carlos, Sorocaba campus, Rodovia João Leme dos Santos, km 110, 18052-780, Sorocaba, Brazil; 4 Institute of Environment & Sustainable Development, Banaras Hindu University, Varanasi, India

## Abstract

The use of lower concentrations and fewer applications of herbicides is one of the prime objectives of the sustainable agriculture as it decreases the toxicity to non-targeted organisms and the risk of wider environmental contamination. In the present work, nanoparticles were developed for encapsulation of the herbicides imazapic and imazapyr. Alginate/chitosan and chitosan/tripolyphosphate nanoparticles were manufactured, and their physicochemical stability was evaluated. Determinations were made of the encapsulation efficiency and release kinetics, and the toxicity of the nanoparticles was evaluated using cytotoxicity and genotoxicity assays. The effects of herbicides and herbicide-loaded nanoparticles on soil microorganisms were studied in detail using real-time polymerase chain reactions. The nanoparticles showed an average size of 400 nm and remained stable during 30 days of storage at ambient temperature. Satisfactory encapsulation efficiencies of between 50 and 70% were achieved for both types of particles. Cytotoxicity assays showed that the encapsulated herbicides were less toxic, compared to the free compounds, and genotoxicity was decreased. Analyses of soil microbiota revealed changes in the bacteria of the soils exposed to the different treatments. Our study proves that encapsulation of the herbicides improved their mode of action and reduced their toxicity, indicating their suitability for use in future practical applications.

The use of chemicals to combat pests and diseases in agriculture is not a recent phenomenon. Substances such as sulfur, arsenic, lime, and nicotine have traditionally been employed for this purpose, although the post-industrial development of the chemical industry has greatly expanded the available range of products used as pesticides. However, inappropriate use of these substances can result in adverse environmental effects^1^.

The herbicides imazapic and imazapyr are the members of the imidazolinone group of compounds mainly used to control weeds in plantations of maize, soybean, and peanut^2^. These compounds can control a broad spectrum of weeds, with effects at extremely low dosages, but a drawback is their high persistence in the soil^3^. The mechanism of action is based on inhibition of the acetolactate synthase enzyme responsible for catalysis of the first step in the synthesis of amino acids such as valine, leucine, and isoleucine, hindering the cellular development of the plant. The compounds are easily transported through the roots and stems. However, the development of resistance by plants has led to the combined use of imazapic and imazapyr, which has been successfully employed in cultivations of maize^4^.

In recent years, there has been an increased interest in nanotechnology as a means of improving the effectiveness of herbicides, while at the same time minimizing their environmental impacts^5^,^6^,^7^,^8^,^9^,^10^. Several studies have described the use of herbicide carrier systems that provide better control of weeds, without harming nontarget organisms^8^,^9^,^10^. Among the various nanostructured systems that can be used with herbicides, some of the most promising are based on polymeric substances^11^,^12^,^13^,^14^,^15^,^16^,^8^,^17^,^9^,^10^. One polymer that has been highlighted for use in the agricultural area is chitosan, a biodegradable substance obtained from the deacetylation of chitin, which is highly effective as a carrier system for agrochemicals and plant micronutrients^18^.

Silva *et al.* (2011) developed a system based on nanoparticles of chitosan and alginate as carriers for the herbicide paraquat. The nanoparticles showed an average diameter of 635 nm, polydispersity index of 0.518, zeta potential of −22.8 mV, and encapsulation efficiency of 74.2%. Encapsulation of the herbicide slowed its release and the results confirmed the effectiveness of using alginate in nanoparticles of chitosan^14^. Grillo *et al.* (2014a) prepared chitosan/tripolyphosphate nanoparticles as a carrier system for paraquat, and found that the encapsulated herbicide was as effective as the free form, but with a slower release profile. The particles showed an average size of 300 nm, encapsulation efficiency of 62%, and low genotoxicity^8^.

Given this background, the aim of the present work was to prepare and characterize nanoparticles based on chitosan (chitosan/alginate – CS/ALG and chitosan/tripolyphosphate – CS/TPP), containing imazapir and imazapyc for use in agricultural weed control. There have been no previous reports of the combination of these herbicides in nanoparticles; therefore the development of effective encapsulation technique represents an important technological advancement in this area.

The formulations were characterized in terms of size distribution, zeta potential, and polydispersity index, as well as their ability to encapsulate the active agents. Comparison was made between the nanoparticle systems and the free herbicides in terms of their cytotoxicity and genotoxicity, in order to confirm that the encapsulation procedure resulted in a reduction in toxicity. The effects of these systems on bacteria associated with the soil nitrogen cycle were evaluated in order to determine the likelihood of any potential impacts on soil microbiota. Finally, tests using a target species (*Bidens pilosa*) was conducted to determine the effectiveness of the encapsulated herbicides, with a view to their use as a safer alternative in agriculture.

## Results and Discussion

### Physicochemical characterization and stability of the formulations

**Figure 1:**
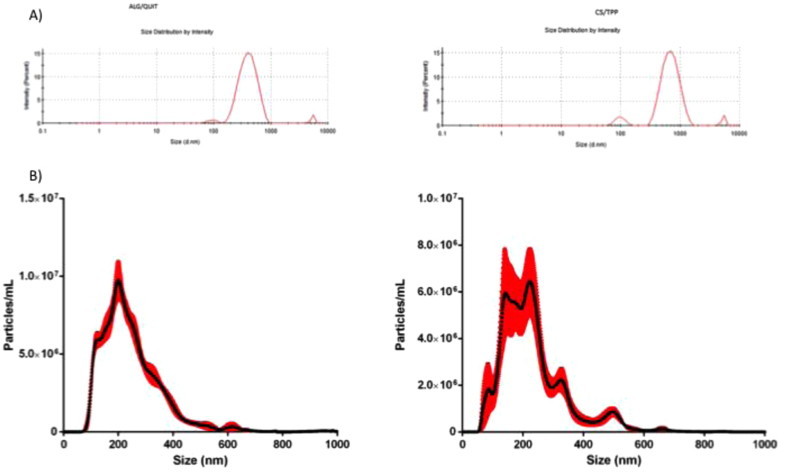
Chemical analysis of the CS/ALG and CS/TPP nanoparticles: (**A**) initial size distributions of the CS/ALG/IMC+IMR and CS/TPP/IMC+IMR formulations; (**B**) number concentrations of the CS/ALG/IMC+IMR and CS/TPP/IMC+IMR nanoparticles as a function of size.

Two different methodologies were used to determine the size distributions of the particles containing the herbicides: (i) dynamic light scattering and (ii) nanoparticle tracking analysis. The average size distributions of the NP-CS/TPP using the DLS and NTA methods were 478.6 ± 52.3 nm and 233.5 ± 10.7 nm, respectively (Fig. 1). The concentration of the CS/TPP nanoparticles containing the herbicides, calculated from the NTA results was 1.20 × 10^9^ particles/mL. The average size distributions of the CS/ALG nanoparticles using the DLS and NTA methods were 377.7 ± 9.7 nm and 246.8 ± 2.6 nm, respectively (Fig. 1). The CS/ALG nanoparticles showed a higher concentration (1.83 × 10^9^ particles/mL) and a size distribution that was more homogeneous (lower polydispersity), compared to the CS/TPP nanoparticles.

Both nanoparticle systems showed more than one particle population. The differences between the data obtained with the two techniques could be explained by the fact that in the NTA technique, the samples were diluted many times, which might have caused the rupture of aggregates, resulting in smaller average size distribution values, compared to the DLS data. Analysis of the CS/TPP and CS/ALG nanoparticles without the presence of the herbicides showed similar size distribution values (data not shown).

**Figure 2:**
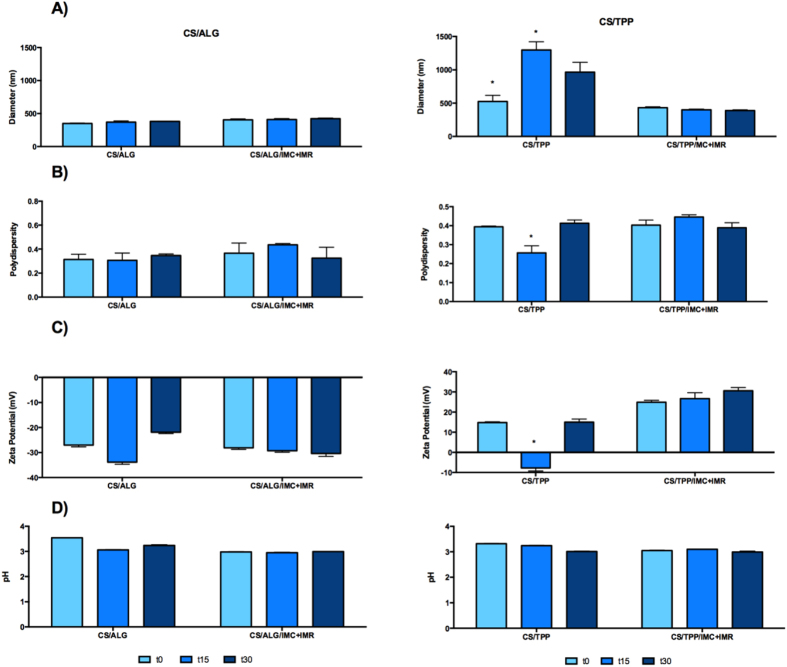
Stability of the nanoparticles over a period of 30 days. CS/ALG: alginate/chitosan nanoparticles; CS/ALG/IMC+IMR: alginate/chitosan nanoparticles containing imazapic and imazapyr; CS/TPP: chitosan/sodium tripolyphosphate nanoparticles; CS/TPP/IMC+IMR: chitosan/sodium tripolyphosphate nanoparticles containing imazapic and imazapyr. (**A**) Size distribution; (**B**) Polydispersity index; (**C**) Zeta potential; (**D**) pH.

The stabilities of the CS/TPP and CS/ALG nanoparticles, with or without the herbicides, were evaluated using measurements of average size distribution, polydispersity, zeta potential, and pH over a period of 30 days (Fig. 2). In the case of the CS/ALG nanoparticles, no significant changes in these parameters were observed, indicating that the particles remained stable (in the presence and absence of the herbicides). An important finding is that the zeta potential remained at around −30 mV, so that electrostatic repulsion was maintained between the particles, minimizing the likelihood of aggregation.

In contrast, in the absence of the herbicides, the CS/TPP nanoparticles showed temporal changes in average size, polydispersity, and zeta potential that were indicative of particle aggregation, which was favored by the low zeta potential (+15 mV). However, the encapsulation of imazapyr and imazapic in the nanoparticles resulted in increased stability, with higher zeta potential (+26 mV). This could be explained by interactions between the herbicides and the polymeric chains of the CS/TPP nanoparticles. Comparison of the stabilities of the two types of nanoparticle containing herbicides showed that the polydispersity of the CS/ALG nanoparticles was lower than that of CS/TPP (Fig. 1).

The encapsulation efficiencies obtained for CS/ALG were 62.3 ± 3.2% and 71.3 ± 2.8% for IMC and IMR, respectively, while the corresponding values for CS/TPP were 58.6 ± 4.5% and 69.6 ± 5.5%, respectively. For both types of nanoparticles, greater encapsulation efficiency was obtained for IMR rather than IMC. In terms of chemical structure, the difference between these compounds is the presence of a –CH_3_ group in the aromatic ring of IMC, which slightly increases the hydrophobicity of this molecule and thereby decreasing the possibility of polar interactions with the polymers of the nanoparticles. Encapsulation efficiencies achieved using CS/ALG nanoparticles have been described in previous work. For instance, Sarmento *et al.* (2007) reported an insulin encapsulation efficiency of around 80%, which was dependent on the relative amounts of CS and ALG^19^. Similarly, Silva *et al.* (2010, 2011) reported encapsulation efficiencies for the herbicides clomazone (60 and 90%)^20^ and paraquat (75%)^14^.

The encapsulation efficiencies of IMC and IMR could be explained by the different strengths of intermolecular forces such as ionic interactions (pKa values of 3.9 for IMC and 3.6 for IMR) and hydrogen bonding (due to the presence of electronegative groups) with the components of the nanoparticles, especially the polymeric chains of chitosan. In the case of the CS/ALG nanoparticles, the presence of two polymers may have facilitated interaction with the herbicides, resulting in greater encapsulation efficiency. Nonetheless, an important point to consider is that the presence of a free herbicide fraction is required in order to provide immediate herbicidal action of the formulation.

### Assays of release of the herbicides from the nanoparticles

**Figure 3:**
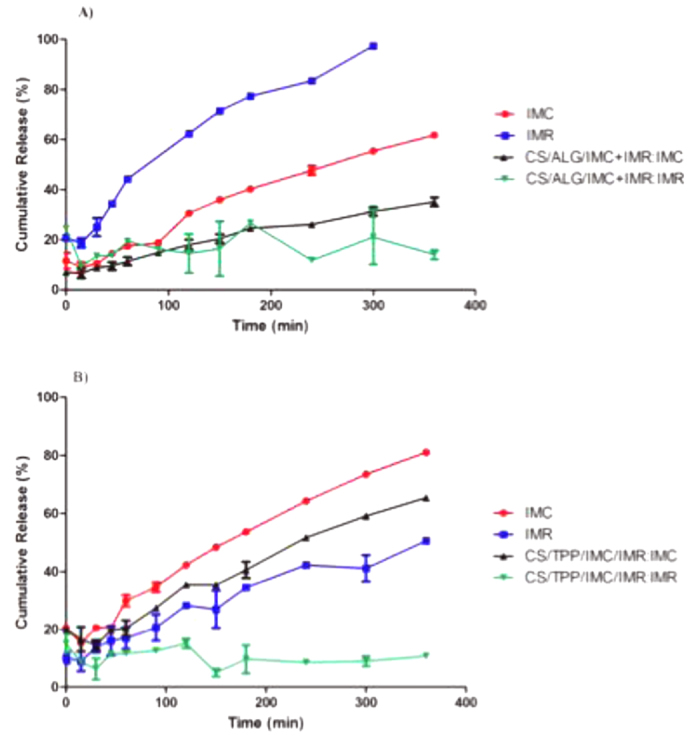
*In vitro* release kinetics profiles for imazapic and imazapyr associated with (**A**) CS/ALG nanoparticles, and (**B**) CS/TPP nanoparticles. Tests performed at 25 ^o^C, in triplicate (n=3), using a system with two compartments (donor and acceptor) separated by a membrane with a molecular exclusion pore size of 1000 Da. The acceptor compartment contained solutions of 0.0022 mol/L calcium chloride (case **A**) or 0.0003 mol/L TPP (case **B**).

The release profiles of the encapsulated combined herbicides from the CS/ALG nanoparticles are shown in Fig. 3. The release percentages of 55 and 97% were obtained for free IMC and IMR, respectively, after 300 min, while in the presence of the nanoparticles, the values decreased to 30% (IMC) and 20% (IMR) after 300 min. The results therefore showed that encapsulation of the combined herbicides resulted in slower release, compared to the free compounds

When the herbicides were encapsulated separately in the CS/TPP nanoparticles, release percentages of 59 and 9% were obtained for IMC and IMR, respectively, after 300 min (Fig. 3). Combined encapsulation of the compounds in the CS/TPP nanoparticles resulted in a substantial decrease (of around 35%) in the release percentage of IMR, compared to separately encapsulated IMR, while the combined encapsulation did not affect the release of IMC.

#### Release mechanisms

**Figure 4:**
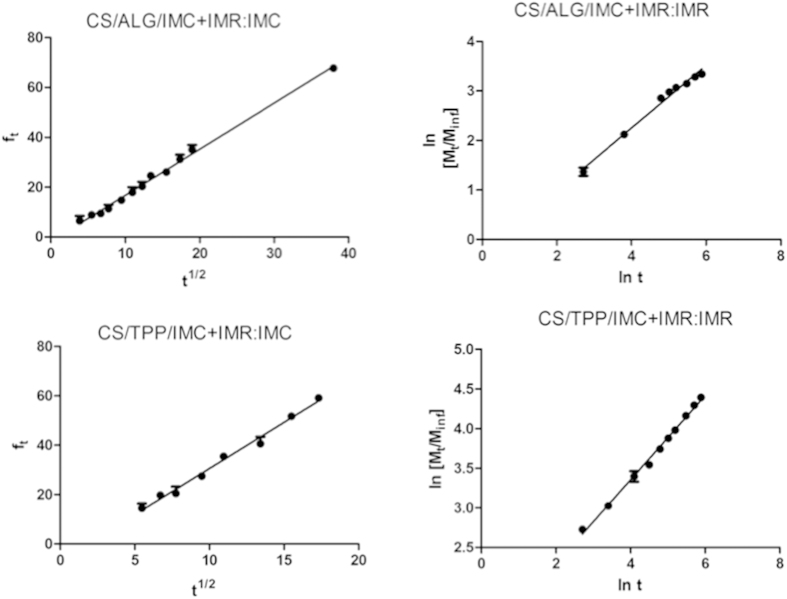
Linearization using the mathematical models that provided the best fits (Higuchi and Korsmeyer-Peppas) for the herbicides IMC and IMR encapsulated individually or in combination in the CS/ALG and CS/TPP nanoparticles.

The Higuchi, Korsmeyer-Peppas, and first order mathematical models were used to investigate the mechanism of release of IMC and IMR encapsulated together in the CS/ALG or CS/TPP nanoparticles. Figure 4 shows the results obtained from linearization of the release curves for IMC and IMR, encapsulated individually or in combination. Linear regression was used to calculate the values of the release constant and release exponent (Table 1). For the herbicides encapsulated together in the CS/ALG nanoparticles, the values obtained indicated that the release of IMC followed a diffusion mechanism according to Fick’s law, while IMR was released by means of Case II transport, reflecting a different mode of interaction between the herbicide and the nanoparticles, as well as the lower encapsulation efficiency of IMR.

Silva *et al.* (2011) found significant differences between the release profiles of free and encapsulated paraquat^14^. The Korsmeyer-Peppas mathematical model was able to describe the behavior of the formulations, and a release coefficient of 0.83 indicated that the release mechanism was by means of non-Fickian diffusion. The release parameter values obtained by fitting of the mathematical models to the data for the CS/TPP nanoparticles (Table 1) showed that the release of IMC was by diffusion following Fick’s law, described by the Higuchi model. The release of IMR obeyed the Korsmeyer-Peppas mathematical model, with a release exponent of 1.23 being indicative of release by relaxation of the polymeric matrix and/or particle dissolution.

Grillo *et al.* (2014a, 2014b) reported an alteration in the release profile of paraquat after encapsulation, and that the release profile of atrazine was modified when the particles were coated with chitosan^8^,^17^. Application of the Korsmeyer-Peppas mathematical model to the release kinetics data gave values for the release constant (k), the release exponent (n), and the correlation coefficient (r) of 1.99 min^−1^, 0.68, and 0.984, respectively. The value of the release exponent, which provides an indication of the type of release mechanism, was in the range 0.43 < n < 0.85, indicating that the process was controlled by anomalous transport (a combination of diffusion and Case II transport).

### Evaluation of nanoparticle toxicity

#### Allium cepa

**Figure 5:**
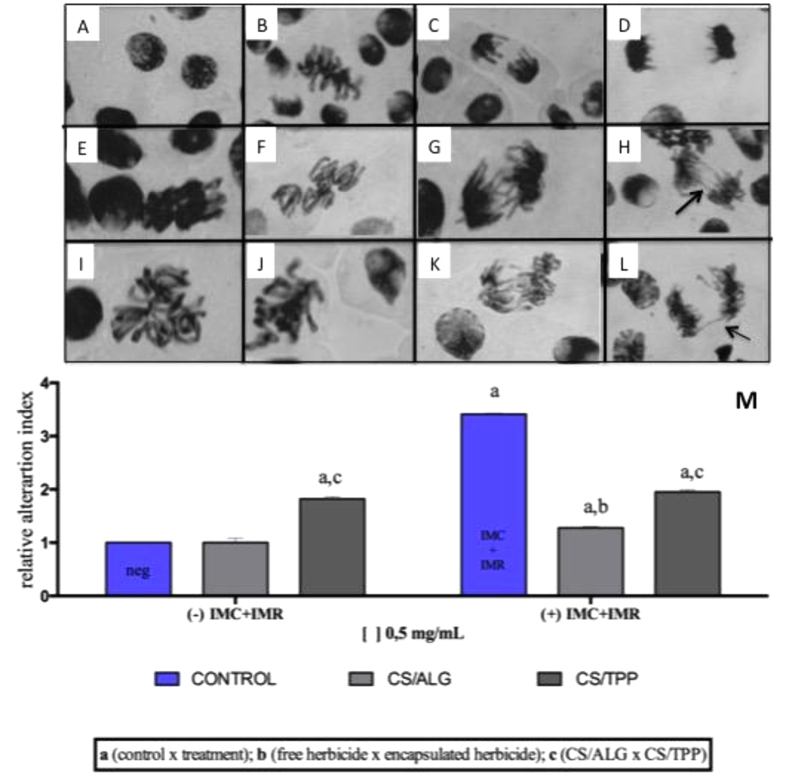
A to D cell division phases: (**A**) prophases; (**B**) metaphases; (**C**) anaphases; (**D**) telophase. In (**E**) to (**L**) show different alterations in division phases. (**M**) Relative alteration index values using the *Allium cepa* assay. The roots were exposed to the free herbicides, the CS/ALG and CS/TPP nanoparticles, and the herbicides encapsulated in the nanoparticles. Experiments performed in triplicate at a concentration of 0.5 mg/mL. The letters a, b, and c indicate significant differences (ANOVA, p < 0.05).

The *Allium cepa* assay provides an indication of the ability of a substance to cause alterations in the genetic material. The results obtained here demonstrated that encapsulation of the herbicides was able to reduce the extent of damage, compared to the free compounds (Fig. 5), indicating that the encapsulation procedure diminished the genotoxicity (the ability to cause alterations in the DNA) of the formulations.

The free herbicides (IMC+IMR) showed a relative alteration index value that was around 100% higher than for the herbicides encapsulated in the CS/ALG or CS/TPP nanoparticles. The lowest alteration index value was found for encapsulation in the CS/ALG nanoparticles. In the absence of the herbicides, the CS/ALG nanoparticles showed the same relative alteration index value as the negative control, while an increase of approximately 50% was observed for CS/TPP. For the encapsulated formulations, a smaller alteration index value was obtained using CS/ALG, which was therefore the best option in terms of reducing genotoxicity. The results therefore showed that encapsulation using CS/ALG provided slower release than the other formulations tested, and that the slower release resulted in less cellular damage over a period of 24 h.

In previous work, Grillo *et al.* (2014a) evaluated the genotoxic potential of free and encapsulated paraquat. The results showed that all the treatments increased DNA damage, compared to the negative control, although the greatest damage was caused by free paraquat, indicating that encapsulation of the herbicide acted to reduce damage to the DNA^8^. In work using atrazine, Grillo *et al.* (2010) showed that its encapsulation in PHBV reduced the toxic effects, compared to the free herbicide, because of the slower release^15^.

#### Comet assays

**Figure 6:**
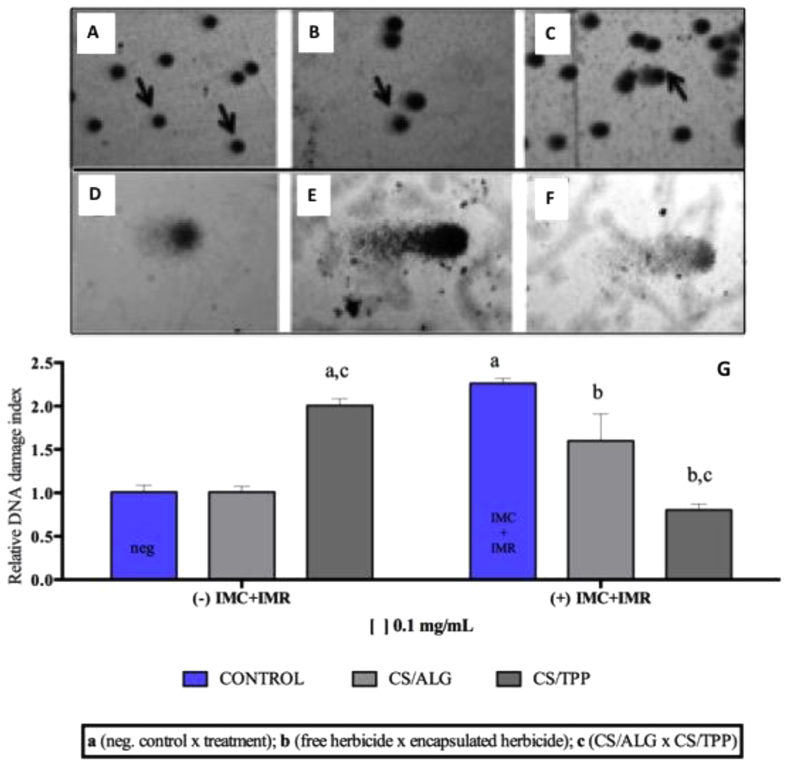
In (**A**) level alteration 0; in (**B**) level alteration 1; (**C**) level alteration 2; (**D**) level alteration 3; (**E**) level alteration 4 and (**F**) apoptosis. In (**G**) Relative damage index values obtained using comet tests with CHO cells exposed to the free herbicides, the CS/ALG and CS/TPP nanoparticles, and the herbicides encapsulated in the nanoparticles, at concentrations of 0.1 and 0.05 mg/mL.

The use of comet assays provides an indication of the capacity of a compound to rupture the genetic material, although it is a pre-mitotic analysis, which means that subsequent repair of the DNA might be possible during division. The comet analysis results showed that the use of the free herbicides resulted in higher damage index values, compared to the herbicides encapsulated in the CS/ALG and CS/TPP nanoparticles (Fig. 6).

The relative damage index value obtained for the CS/ALG nanoparticles without herbicides was similar to that for the control. However, in the case of the CS/TPP nanoparticles, the value obtained was higher than for both the CS/ALG nanoparticles and the negative control. The free herbicides showed the highest damage index value, which was 50% higher than that of the control. Encapsulation of the herbicides in the CS/TPP or CS/ALG nanoparticles reduced the extent of damage, with the best results obtained for CS/TPP+herbicides, for which the index value was the same as that for the negative control. Nonetheless, no significant differences in genotoxicity were found between the negative control and the herbicides encapsulated using the two types of nanoparticle.

Similar findings were reported in previous *in vivo* studies using the same herbicides, with low toxicity to rats, dogs, and rabbits^2^. Grillo *et al.* (2012) compared DNA damage caused by treatments with triazine herbicides, free or encapsulated in poly-epsilon-caprolactone nanocapsules, and found that encapsulation resulted in reduced DNA damage^16^. Lima *et al.* (2012) studied the herbicide ametryn and found that encapsulation in microparticles of poly-hydroxybutyrate (PHB) and poly-hydroxybutyrate-co-hydroxyvalerate (PHBV) reduced DNA damage^21^.

### Analysis of the effects of the herbicides and nanoparticles containing the herbicides on soil microbiota

Quantification of the bacterial genes responsible for the production of enzymes active in the soil nitrogen cycle enabled assessment of the effects of the herbicides in their free and encapsulated forms on the numbers and proportions of bacteria responsible for different stages of the cycle. This information is important, because a properly functioning nitrogen cycle is essential for soil fertility. The genes studied in the present work corresponded to nitrogen fixing, nitrifying, and denitrifying bacteria. Many studies have shown associations between environmental conditions, changes in the microbial community, and soil characteristics, considering aspects including contaminants, nutrients, water availability, and type of soil, amongst others^22^,^23^. Genes have been used to evaluate the cycles of nitrogen, carbon, phosphorus, and sulfur^24^,^25^,^26^,^27^.

**Figure 7:**
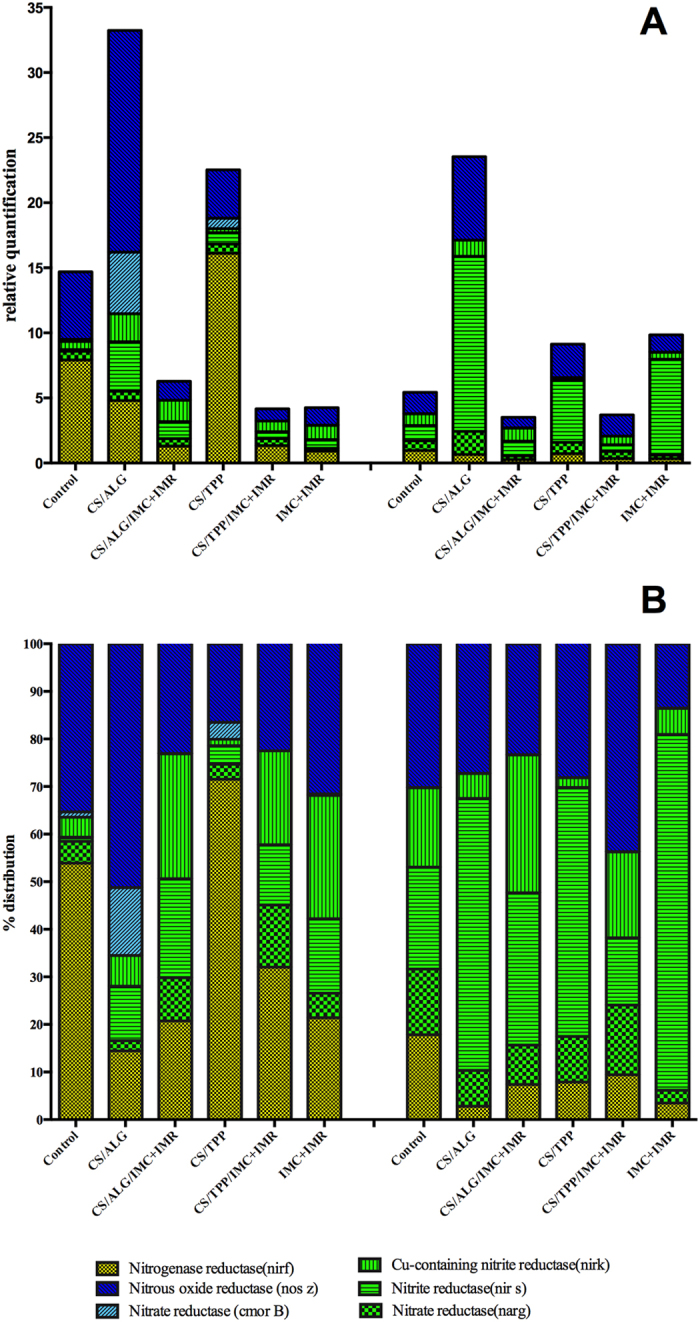
Quantitative soil analyses using the *nirF*, *nosZ*, *cmorB*, *nirK*, *narG*, and *nirS* genes: (**A**) 7 days after treatment; (**B**) 30 days after treatment. Proportions of genes present in the soil samples: (**A**) 7 days after treatment; (**B**) 30 days after treatment.

Studies of the soil microbiota and their activities contribute to understanding the structure of the microbial community and the enzymatic activity involved in the cycles of elements in the soil. The present work focused on quantification of certain bacterial genes responsible for the synthesis of enzymes related to the nitrogen cycle. The evaluation was made relative to an initial control soil and a negative control soil that was monitored until the end of the experiment. Both of these soils remained untreated. The results revealed an increase in the numbers of bacteria in the soils treated with nanoparticles (CS/ALG and CS/TPP) (Fig. 7). This was mainly evident 7 days after application of the treatments, with an increase in the number of bacteria possessing the gene for synthesis of the nitrate reductase enzyme. Treatment of the soil with the free and encapsulated herbicides resulted in decreases in bacterial numbers, with the least alterations, compared to the negative control, shown by the soil exposed to the CS/ALG/IMC+IMR formulation. After 30 days, higher numbers of bacteria (compared to the negative control) were observed for the soils treated with the nanoparticles alone (CS/ALG and CS/TPP), while the treatment with CS/ALG/IMC+IMR showed the greatest similarity to the negative control.

Calculation of the percentage fractions of the genes related to the nitrogen cycle were used to determine the proportions of the different bacterial types present (Fig. 7B). The bacteria responsible for reduction of NO to N_2_O by the nitrate reductase enzyme constituted the largest fraction in the treatment with CS/ALG, while treatment with CS/TPP resulted in the greatest increase in the proportion of bacteria that produce the nitrogenase reductase enzyme responsible for the fixation of atmospheric nitrogen. This finding can be explained by the chemical composition of the nanoparticles, because the phosphate in TPP is likely to have a positive effect on bacterial growth, hence facilitating the process of nitrogen fixation^28^. The proportions of bacteria in the soil treated with the free herbicides were similar to those of the negative control, although the absolute numbers of bacteria were lower.

After 30 days, the treated soils showed distributions of bacteria similar to that of the control soil, with recovery shown by the soil treated with the herbicides (IMC+IMR), in terms of the total number of bacteria. However, there was a general increase in the number of bacteria responsible for denitrification, especially the first stage (nitrite and nitrate reductases – *nirK*, *nirS*, and *narG* genes), as well as the second stage (nitrate reductase and nitric oxide reductase – *cnorB* and *nosZ* genes), although the numbers of bacteria involved in the second step were smaller for the herbicide-treated soils. When the concentration of denitrifying bacteria involved in Stage 1 is smaller than the concentration for Stage 2, there is likely to be release of nitrogen to the environment. An abundance of bacteria presenting the copper-containing nitrite reductase gene (*nirK*), together with those presenting the nitrite reductase gene (*nirS*), results in increased degradation of NO_2_^−^ and greater NO formation.

It should be noted that the nanoencapsulation systems altered the release profiles of the herbicides, with slower release during the first 7 days, so that the herbicidal activity outside the nanoparticles was lower, compared to the high initial level of activity provided by the free herbicides.Changes over time in the bacterial community responsible for the soil nitrogen cycle can be caused by physical factors such as heat, water availability, and pH^29^,^30^,^31^,^32^,^33^,^34^. Wang *et al.* (2014) used the qPCR technique and specific genes associated with the soil nitrogen cycle to identify alterations in the soil microbiota and ecological processes in different types of fertilized soils^33^.

The alterations observed up to 30 days indicated that imazapic and imazapyr induced changes in the soil microbiota in terms of both the quantity and types of the bacteria involved in the soil nitrogen cycle, with a decrease in the proportion of nitrogen-fixing organisms and an increase in denitrifying bacteria (especially those involved in the first step of denitrification). The soils treated with the encapsulated herbicides showed different microbiota profiles, compared to soils treated with the free herbicides. The bacterial profiles of the soils treated with the CS/ALG/IMC+IMR and CS/TPP/IMC+IMR formulations showed similarity to the negative control, especially in the case of CS/ALG/IMC+IMR. This indicated that the carrier systems employed in the present work acted to reduce changes in the soil nitrogen cycle bacteria, compared to use of the free herbicides.

### Evaluation of herbicidal activity

**Figure 8:**
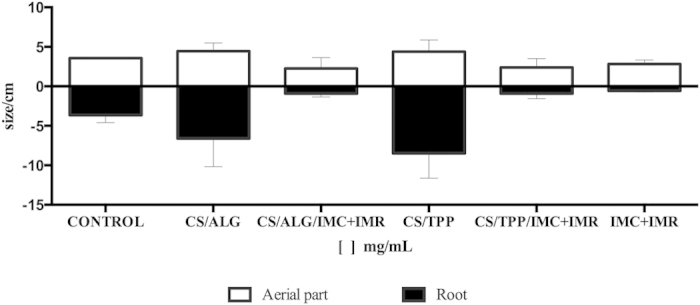
Effects of the CS/ALG and CS/TPP nanoparticles and the encapsulated herbicides (CS/ALG/IMC+IMR and CS/TPP/IMC+IMR), at concentrations equivalent to 400 g/ha, on the growth of *Bidens pilosa*.

The results described above demonstrated that encapsulation of the herbicides in the CS/TPP and CS/ALG nanoparticles led to a reduction in toxicity, indicating that the formulations could be attractive options for use in agriculture. The effectiveness of the encapsulated herbicides was therefore tested against a target weed species, *Bidens pilosa* (black-jack). In this assay, the parameters evaluated were the fresh masses of the roots and aerial parts of the plants, and the herbicides were applied at a dosage equal to that used in the field (400 g/ha). Treatment using both the free and encapsulated herbicides resulted in reduced growth, compared to the control (Fig. 8). The findings indicated that encapsulation should result in a reduction of toxicity of the herbicides towards nontarget organisms, while maintaining adequate herbicidal activity. Similarly, Grillo *et al.* (2014a) found that plants treated with CS/TPP nanoparticles containing paraquat showed lower growth than plants that received the free herbicide^8^.

## Conclusions

The results of this work showed that CS/ALG and CS/TPP nanoparticles were able to encapsulate the herbicides imazapic and imazapyr with efficiencies exceeding 60%. Release kinetics experiments demonstrated that the encapsulated herbicides were released more slowly than the free forms. In the case of imazapic, the mechanism of release from both types of nanoparticle was by simple diffusion, while imazapyr was released from the CS/ALG nanoparticles by anomalous transport and from the CS/TPP nanoparticles by relaxation of the polymeric matrix. The nanoparticle size was smaller than 400 nm, the polydispersity index was around 0.3, and the zeta potentials of the formulations employing CS/ALG and CS/TPP were −30 and +26 mV, respectively. The formulations remained stable for 30 days.

Cytotoxicity assays indicated that the nanoparticle systems presented low toxicity. Genotoxicity assays using different cell types (CHO and *Allium cepa*) showed that the encapsulated herbicides caused less damage, compared to the free compounds. Tests using soil microbiota revealed that the free and encapsulated herbicides did not substantially affect the total numbers of bacteria present in the soil. However, molecular analysis using specific genes showed that there were changes in the amounts and types of bacteria associated with the soil nitrogen cycle, with the least interference shown by the herbicides encapsulated in the CS/ALG nanoparticles.

In tests of herbicidal activity, the encapsulated compounds were found to be more effective than the free forms, enabling the use of smaller dosages. In terms of reduced toxicity and herbicidal effectiveness, the best results were obtained using the CS/ALG nanoparticles. This formulation therefore offers an effective alternative for weed control in agricultural cultivations. Further tests are underway to evaluate the toxicity and herbicidal effectiveness of formulations containing lower concentrations of the herbicides.

The present study underlines that the encapsulation of two herbicides in one carrier system to improve the activity and reduces the impacts to humans and environment is a good strategy for the sustainable agricultural production. However, more research in this field is need in order to scale-up this technology for largescale applications and also for underpinning the mode of action of these nanopesticides in plants at molecular level.

## Methods

### Preparation of the nanoparticles

#### Alginate/chitosan nanoparticles

The alginate/chitosan (CS/ALG) nanoparticles were manufactured using the ionotropic gelification method described by De and Robinson (2003)^35^. A 10 mL solution of sodium alginate was prepared, to which was added the herbicides imazapic (IMC) and imazapyr (IMR). A solution of calcium chloride (0.24 mg/mL) was then added dropwise to the first solution. The resulting calcium alginate pre-gel was maintained under agitation for 30 min. Subsequently, a solution of chitosan (0.24 mg/mL) in 1% acetic acid, previously prepared and kept under agitation for 12 h to solubilize the polymer was added and the mixture was kept under agitation overnight to enable the formation of nanospheres. The final concentrations of imazapyr and imazapic in the formulation were 1 g/mL.

#### Chitosan/tripolyphosphate nanoparticles

For the production of chitosan/tripolyphosphate (CS/TPP) nanoparticles, a 0.2% solution of chitosan in 0.6% acetic acid was prepared and kept under agitation for 12 h. The solution was then diluted to 0.1% chitosan in 0.3% acetic acid, and the herbicides were added. The final solution volume was 10 mL. In the next step, 6 mL of a 0.1% solution of TPP (at pH 4.5 and 8 ^o^C) were added dropwise to the chitosan solution and the mixture was agitated for 12 h. The final concentrations of imazapir and imazapic in the formulation were 1 mg/mL.

### Physicochemical characterization

Characterization of the CS/TPP and CS/ALG nanoparticles was performed by means of size distribution measurements using dynamic light scattering (DLS) and nanoparticle tracking analysis (NTA). The DLS analyses employed a ZS90 instrument (Malvern Instruments, UK). The NTA measurements used 1 mL of nanoparticle suspension, with each measurement consisting of five repetitions with 90 s videos and an average of 100 particles per 1 s frame. The result was a graph of concentration according to nanoparticle size. All the DLS and NTA analyses were made in triplicate. The polydispersity and zeta potential of the nanoparticles were determined by microelectrophoresis, using a Zeta Plus analyzer (Malvern Instruments). The stability of the suspensions, with and without the herbicides, was evaluated by measuring the average particle diameter, polydispersity, and zeta potential during a period of 30 days, with the samples stored in amber flasks at ambient temperature (25 °C).

### Efficiency of encapsulation of the herbicides in the nanoparticles

The total amount (100%) of herbicide present in the polymeric nanoparticle suspension was determined by dilution of the suspensions in methanol. This solution was filtered through a 0.22 μm membrane (Millipore), and quantification was performed using high performance liquid chromatography (HPLC). The amounts of herbicides encapsulated in the nanoparticles were determined using the ultrafiltration/centrifugation method, with the nanoparticle suspensions centrifuged in 30 kDa regenerated cellulose ultrafiltration devices (Microcon, Millipore). In this procedure, only the herbicides were able to cross the membrane, and the ultrafiltrate was analyzed by HPLC. The amounts of herbicide associated with the nanoparticles could then be obtained from the difference between the total amounts (100%) and the amounts not associated with the nanoparticles^36^,^37^,^38^.

### Release kinetics assays

Assays to measure the release profiles of the herbicides were performed using a system with two compartments (donor and acceptor), maintained under gentle agitation^15^. A cellulose membrane (1 kDa, Spectrapore) was used to separate the sample (2 mL) in the donor compartment from the acceptor compartment. The pores of the membrane only allowed passage of the free herbicides, with the herbicides associated with the nanoparticles being retained. Samples were collected from the acceptor compartment for HPLC analysis after different time intervals. All the measurements were performed in triplicate. The mechanisms of release of the herbicides were evaluated using the first order, Higuchi, and Korsmeyer-Peppas mathematical models^39^,^40^,^41^.

### Cytotoxicity and genotoxicity assays

#### Allium cepa assays

In these tests, the roots of germinated seeds were placed for 24 h in the different media (containing nanoparticles, nanoparticles with herbicides, and herbicides, at concentrations of 0.5 mg/mL). After this period, the roots were fixed in methanol:acetic acid (3:1) for 24 h, followed by treatment with 1 mol/L HCl for 9 min at 60 °C. The samples were dyed with Schiff reagent for 2 h and then spread onto slides, with the addition of one drop of 2% acetic-carmine. The slides were examined using an optical microscope at a magnification of x40. The results were used to calculate the mitotic index (MI) and the damage index (DI).

#### Comet tests

Comet tests were performed using Chinese hamster ovary (CHO) cell cultures. The CHO cell line was maintained in bottles containing McCoy culture medium (Gibco) supplemented with 10% fetal bovine serum and 1% antibiotic, kept in a heating cabinet at 37 °C under a humid atmosphere with 5% CO_2_, until reaching a satisfactory number of cells for use in the assays. The cells were then subcultured and subsequently transferred to 6-well plates, followed by treatment during 1 h with the CS/ALG and CS/TPP nanoparticles, with and without the herbicides. Negative controls were included, without any treatment. After a period of 1 h, the cells (treated and controls) were separately mixed with low melting agarose (0.8%) at 37 °C, and the mixture was spread onto a slide that had been pre-coated with normal agarose (1.5%). Cover slips were placed over the samples and the slides were refrigerated until the agarose had solidified. The cover slips were then removed and the slides were submerged in lysis solution for 1 h, followed by 5 min in neutralization solution (Tris).

After neutralization, the slides were transferred to a horizontal electrophoresis cuvette containing buffer and allowed to rest for 20 min before starting the run, which was performed for 20 min. The slides were again neutralized for 10 min, then washed in water for 5 min and dried overnight at ambient temperature. The samples were subsequently rehydrated, dried again in a drying cabinet at 37 °C, transferred to fixing solution (containing trichloroacetic acid, zinc sulfate, and glycerol) for 10 min, and dried at 37 °C. In the next step, the slides were hydrated for 5 min in distilled water, stained with silver nitrate for 30 min, placed in stop solution (acetic acid) for 5 min, and washed three times with distilled water. The final drying was performed at ambient temperature, and the comet analysis employed an optical microscope at x40 magnification, with around 100 randomly selected cells examined for each sample.

The damage indices were calculated according to the sizes of the tails. The tails were scored in five levels (0, 1, 2, 3 and 4), where zero and four represent respectively the lower and higher comet tails size (Eq1). A specialist analyzed the slides using a single-blind-review in order to minimize variability.


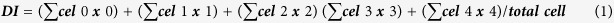


### Analysis of the effects of the herbicides and the herbicide-loaded nanoparticles on soil microbiota

The soil used in this experiment was obtained from a local agricultural supplier. The chemical composition of the soil was characterized using X-ray fluorescence, considering the elements aluminum, calcium, chlorine, iron, potassium, magnesium, phosphorus, silicon, sulfur, titanium, chromium, manganese, zinc, strontium, yttrium, zirconium, arsenic, and bromine (Table 2). A 30 g quantity of soil was weighed and sieved for each treatment (negative control, CS/ALG, CS/ALG/IMC+IMR, CS/TPP, CS/TPP/IMC+IMR, and IMC+IMR). A single exposure to the test media was performed, after which the soils were watered twice weekly in order to maintain constant humidity.

The soil was placed in containers, with surface area of 0.025 m^2^, and the quantities of herbicides used were equivalent to the application rates employed in the field, with a proportion of 75% imazapic to 25% imazapyr (using concentrations of 75 g/L of imazapic and 25 g/L of imazapyr). Extraction and analysis of the soil microbiota DNA was conducted 7 and 13 days after application of the treatments.

### Molecular analysis of the soil microbiota

#### Extraction of DNA from the soil

DNA extraction for the polymerase chain reactions employed a PowerSoil DNA Extraction Kit. The genetic material was quantified by fluorescence, using a Qubit 3.0 Fluorometer and a Qubit dsDNA BR Assay Kit (Invitrogen). After quantification, the extractions were diluted to a final total DNA concentration of 1000 ng/mL.

#### qPCR

Quantification of the 16S rRNA*, nifH*, *nirk*, *nirS*, *narG (Escherichia coli)*, *norB*, *nosZ*, *and narG* (*Pseudomonas aeruginosa*) bacterial genes in the soil samples treated with the nanoparticles (with and without herbicides) and the free herbicides was achieved using real-time polymerase chain reactions (qPCR). The primers employed are described in Table 3. The reactions were performed in a final volume of 25 μL containing: 12.5 μL of Platinum SYBR Green qPCR SuperMix-UDG with ROX (Invitrogen), 1 μL of each primer (sense and anti-sense), 1 μL of template (DNA previously extracted from the soil using a PowerSoil DNA Isolation Kit (MO BIO Laboratories, Inc.), and autoclaved ultrapure water to make up the volume to 25 μL. The amplification followed the procedure described by Jung *et al.* (2011), with an initial denaturation at 95 °C for 3 min, followed by 40 cycles of 95 °C for 45 s, 60 °C for 45 s, and 72 °C for 45 s. The fluorescence was measured at the end of each incubation at 60 °C^42^.

A calibration curve was constructed using serial dilutions of the DNA (1:1, 1:10, 1:100, and 1:1000 v/v) and the slope was used to calculate the average amplification efficiency. The sample quantification results were expressed as ΔΔCT, comparing the control and the treated samples.

### Evaluation of herbicidal activity

The activities of imazapic and imazapyr (free or encapsulated), were evaluated using *Bidens pilosa* (black-jack) in 10 cm diameter pots. The soil used was a commercial product composed of pine bark, peat, vermiculite, class A organic agricultural waste, wood sawdust, manure, bone meal, magnesium thermophosphate, and castor oil cake. The experiment was performed in triplicate, using the free and encapsulated herbicides (IMC+IMR), the two types of nanoparticle (CS/ALG and CS/TPP) without herbicides, and untreated controls. Ten *Bidens pilosa* seeds were planted in each pot, totaling 30 seeds per triplicate. The quantities of herbicide used were equivalent to the amounts used in the field (400 g/ha), with the treatments applied pre-emergence. The pots were filled with around 200 g of substrate, the seeds were planted, and the treatments were applied (IMC, IMR, CS/ALG, CS/ALG/IMC+IMR, CS/TPP, and CS/TPP/IMC+IMR). The pots were kept in a greenhouse and were watered every day (morning and afternoon) during 15 days. After this period, the plants were removed and the following parameters were analyzed: germination efficiency, height, root length, and green mass.

## Additional Information

**How to cite this article**: Maruyama, C. R. *et al.* Nanoparticles Based on Chitosan as Carriers for the Combined Herbicides Imazapic and Imazapyr. *Sci. Rep.* **6**, 19768; doi: 10.1038/srep19768 (2016).

## Figures and Tables

**Table 1:** Parameter values obtained by fitting of mathematical models to the data for the CS/ALG and CS/TPP nanoparticles containing herbicides.

Parameter	Korsmeyer-Peppas	Higuchi	First order
CS/ALG/IMC+IMR:IMC			
Release constant (k)	1.689 min^−1^	1.760 min^−1^	2.13 × 10^−3^ min^−1^
Release exponent (n)	0.5564	–	–
Correlation coefficient (r)	0.9892	0.9957	0.8920
CS/ALG/IMC+IMR:IMR			
Release constant (k)	1.578 min^−1^	11.08 min^−1^	2.78 × 10^−3^ min^−1^
Release exponent (n)	1.283	–	–
Correlation coefficient (r)	0.9877	0.4819	0.9079
CS/TPP/IMC+IMR:IMC			
Parameter	Korsmeyer-Peppas	Higuchi	First order
Release constant (k)	7.800 min^−1^	6.789 min^−1^	1.82 × 10^−3^ min^−1^
Release exponent (n)	1.671	–	–
Correlation coefficient (r)	0.1684	0.9897	0.9607
CS/TPP/IMC+IMR:IMR			
Parameter	Korsmeyer-Peppas	Higuchi	First order
Release constant (k)	1.881 min^−1^	7.407 min^−1^	1.95 × 10^−4^ min^−1^
Release exponent (n)	1.230	–	–
Correlation coefficient (r)	0.9948	0.3559	0.1948

The models providing the best fits to the release profiles are shown in bold type.

**Table 2:** Analysis of soil composition using X-ray fluorescence.

Element	Concentration (mg/cm^2^)	Element	Concentration (mg/cm^2^)
Aluminium	0.832	Titanium	0.755
Calcium	1.675	Chromium	0.004
Chloro	0.174	Manganese	0.029
Iron	5.654	Zinc	0.007
Potassium	0.454	Strontium	0.008
Magnesium	0.252	Ytrium	0.002
Phosphurus	0.004	Zirconium	0.087
Silicon	15.007	Arsenium	0.000
Sulfur	0.049	Bromine	0.000

**Table 3:** Primers used in the qPCR reaction for quantification of specific genes.

Gene	Primer	Sequence	pb	Reference
*Bacterial 16S rRNA gene*	341F 534R	5′ CCTACGGGAGGCAGCAG 3′ 5′ ATTACCGCGGCTGCTGGCA 3′	193	Watanabe *et al.*, 2001^43^
*Nitrogenase reductase; nifH*	nifHF nifHRb	5′AAAGGYGGWATCGGYAARTCCACCAC 3′ 5′TGSGCYTTGTCYTCRCGGATBGGCAT 3′	400	Rösch and Bothe, 2005^44^
*Cu-containing nitrite reductase; nirK*	nirK 1F nirK 5R	5′GGMATGGTKCCSTGGCA 3′ 5′GCCTCGATCAGRTTRTGGTT 3′	514	Braker *et al.*, 1998^45^
*Nitrite reductase; nirS*	nirS cd3AF nirS R3cd	5′GTSAACGTSAAGGARACSGG 3′ 5′GASTTCGGRTGSGTCTTGA 3′	425	Throback *et al.*, 2004^46^
*Nitrate reductase; norB*	cnorB2F cnorB6R	5′GACAAGNNNTACTGGTGGT 3′ 5′GAANCCCCANACNCCNGC 3′	389	Braker and Tiedje, 2003^47^
*Nitrous oxide reductase; nosZ*	nosZ-F nosZ-R	5′CGYTGTTCMTCGACAGCCAG 3′ 5′CGSACCTTSTTGCCSTYGCG 3′	453	Kloos *et al.*, 2001^48^
*Nitrate reductase; narG*	narG-f narG-r	5′TCGCCSATYCCGGCSATGTC 3′ 5′GAGTTGTACCAGTCRGCSGAYTCSG 3′	173	Bru *et al.*, 2007^49^
