# Corrigendum: Nanoparticles Based on Chitosan as Carriers for the Combined Herbicides Imazapic and Imazapyr

**DOI:** 10.1038/srep23854

**Published:** 2016-04-13

**Authors:** Cintia Rodrigues Maruyama, Mariana Guilger, Mônica Pascoli, Natalia Bileshy-José, P. C. Abhilash, Leonardo Fernandes Fraceto, Renata de Lima

Scientific Reports 6: Article number: 1976810.1038/srep19768; published online: 01272016; updated: 04132016

This Article contains errors.

In the Methods section under subheading ‘Analysis of the effects of the herbicides and the herbicide-loaded nanoparticles on soil microbiota’,

“The chemical composition of the soil was characterized using X-ray fluorescence, considering the elements aluminum, calcium, chlorine, iron, potassium, magnesium, phosphorus, silicon, sulfur, titanium, chromium, manganese, zinc, strontium, yttrium, zirconium, arsenic, and bromine (Table 2).”

should read:

“The chemical composition of the soil was characterized using X-ray fluorescence (University of Sorocaba multiuser laboratory of Applied Nuclear Physics – Lafinau), considering the elements aluminum, calcium, chlorine, iron, potassium, magnesium, phosphorus, silicon, sulfur, titanium, chromium, manganese, zinc, strontium, yttrium, zirconium, arsenic, and bromine (Table 2).”

In Table 2, ‘mg/cm^2^’ should read ‘wt. (%)’. In addition, the error values were omitted. The correct Table 2 appears below as Table 1.

In addition, the Acknowledgements section is incomplete.

“The authors would like to thank the following Brazilian funding agencies: São Paulo State Science Foundation (FAPESP, grants 2013-12322-2, 2013-07002-9, and 2014/21618-5), CNPq (grant 470529/2013-0), CAPES, and FUNDUNESP.”

should read:

“The authors would like to thank the following Brazilian funding agencies: São Paulo State Science Foundation (FAPESP, grants 2013-12322-2, 2013-07002-9, and 2014/21618-5), CNPq (grant 470529/2013-0), CAPES, and FUNDUNESP. Also the authors would like to thank Prof. José Martins de Oliveira Junior (University of Sorocaba) to X-ray fluorescence soil sample analysis and to the University of Sorocaba Multiuser Laboratory of Applied Nuclear Physics - Lafinau (supported by São Paulo Science Foundation - grant# 2012-15651-4).”

## Figures and Tables

**Table 1:** 

Element	wt. (%)	Element	wt. (%)
Aluminium	0.832 ± 0.398	Titanium	0.755 ± 0.048
Calcium	1.675 ± 0.036	Chromium	0.004 ± 0.000
Chloro	0.174 ± 0.120	Manganese	0.029 ± 0.002
Iron	5.654 ± 0.049	Zinc	0.007 ± 0.007
Potassium	0.454 ± 0.026	Strontium	0.008 ± 0.000
Magnesium	0.252 ± 1.008	Ytrium	0.002 ± 0.000
Phosphurus	0.004 ± 0.025	Zirconium	0.087 ± 0.001
Silicon	15.007 ± 1.905	Arsenium	0.000 ± 0.000
Sulfur	0.049 ± 0.032	Bromine	0.000 ± 0.000

